# Contemporary enteral and parenteral nutrition before surgery for gastrointestinal cancers: a literature review

**DOI:** 10.1186/s12957-018-1393-7

**Published:** 2018-05-16

**Authors:** Michal Jankowski, Manuela Las-Jankowska, Massaoud Sousak, Wojciech Zegarski

**Affiliations:** 1 Surgical Oncology, Ludwik Rydygier Collegium Medicum in Bydgoszcz, Nicolaus Copernicus University in Torun, Oncology Centre, Bydgoszcz, 2 Romanowskiej Str, 85-796 Bydgoszcz, Poland; 2 Department of Surgical Oncology, Oncology Center – Prof Franciszek Lukaszczyk Memorial Hospital, Romanowskiej, Bydgoszcz, Poland; 3 Department of Clinical Oncology, Oncology Center – Prof Franciszek Lukaszczyk Memorial Hospital, Bydgoszcz, Poland; 4 Department of General Surgery, Paluki Health Centre, Znin, Poland

## Abstract

**Background:**

Gastrointestinal cancers are among the most recognised oncological diseases in well-developed countries. Tumours located in the digestive tract may cause the fast occurrence of malnutrition.

**Main text:**

The perioperative period is a special time for systemic metabolism. Thanks to published guidelines, early universal control nutritional status before treatment, patients may have a chance to get suitable nutritional intervention. Although the first line of the intervention—nutritional consultation as well as the fortification of a diet and oral nutritional support (ONS)—is not debatable, in a case of inability of undergoing an oral feeding, the choice of the way of administration in patients before a surgery may represent a serious clinical obstacle.

**Conclusions:**

Although there is broad agreement in the staging, classification, and role of surgery and nutritional status for outcomes of treatment of gastrointestinal cancers, there the way of nutritional intervention in patients with gastrointestinal cancer are still discussed.

## Background

Gastrointestinal cancers are among the most recognised oncological diseases in well-developed countries. In 2010 in Poland, there were 30,382 cases of cancer of the upper and lower gastrointestinal tract with pancreatic cancer, liver cancer and bile duct cancer [1]. Despite the fact that malnutrition is mostly accompanied by the advanced malignant disease, even small tumours located in the digestive tract may cause the occurrence of deep malnutrition—e.g. in case of oesophagus, stomach, and pancreatic cancer. In patients with these neoplasms, malnutrition is the most recognised among all of oncology patients. Hébuterne et al. published the results of a 1-day survey conducted in 154 French hospitals, which involved 1903 patients with malignant disease. Malnutrition was recognised in 39% of all patients, respectively: 67%—pancreatic cancer, 60%—oesophagus cancer and 39%—colon cancer [2].

## Main text

### Malnutrition and surgery

The nutrition of a patient with cancer is one of the basic factors which impacts on the safety of a surgery. Malnutrition has a significant impact on increasing the risk of postoperative mortality and appearance of complications, and this impact increases with the degree of malnutrition [3, 4]. Due to the frequency of malnutrition in patients with malignant gastrointestinal cancer, their state of nutrition is of crucial importance for the results of treatment. Wan Hu published the results of a retrospective study involving 42,483 patients with colorectal cancer based on the register of the American College of Surgeons National Surgical Quality Improvement Program [5]. The researchers were comparing patients categorised based on albuminemia before a surgery (< 3.5 g/dL), weight loss (10% for 6 months before a surgery) and BMI (below 18.5/18.5–29.9). Taking into account a number of factors, malnutrition has been statistically confirmed to have significant impact on increased 30-day morality rate and also impaired wound healing, the occurrence of postoperative pneumonia, re-intubation in the postoperative period, the occurrence of sepsis and septic shock, more frequent transfusions of blood derivative products, and more frequent deep vein thrombosis.

### ESPEN guidelines, National Guidelines

Widely accepted guidelines for nutritional interventions are based on ESPEN (the European Society for Clinical Nutrition and Metabolism) recommendations, which were published separately for enteral and parenteral nutrition in 2006 and 2009 [6, 7]. New recommendations for surgery and oncology are published in 2016 and 2017 [8, 9]. Nutritional intervention before major surgery in a situation of diagnosis in one of the severe nutritional risks (Table 1) or in patients with malnutrition should be undertaken for the minimum of 10 to 14 days, even for the price of the delay of surgical intervention (minimum 7–14 days), and it is recommended with the highest reference level (strong recommendation). Enteral nutrition is the preferred way of feeding. Parenteral nutrition is only recommended when a patient cannot be fed via the digestive tract.

**Table 1:** Severe nutritional risk for surgical patient in preoperative period—ESPEN [6–9]

Weight loss > 10–15% within 6 months	
BMI < 18.5 kg/m^2^	
Subjective global assessment, grade C or NRS > 5	
Serum albumin < 30 g/L (with no evidence of hepatic or renal dysfunction)	

**Fig. 1:**
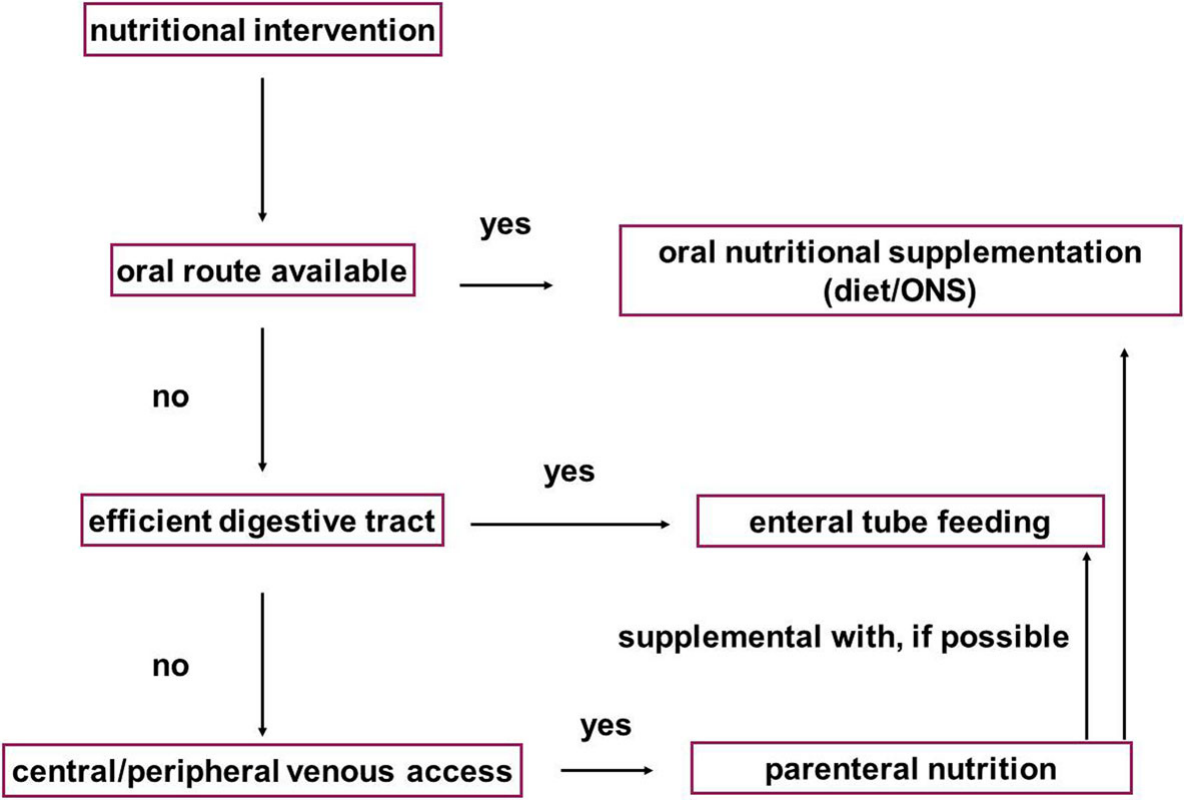
General principles of nutritional intervention [31]

National recommendations were published in several countries. Polish recommendations on the clinical nutrition in oncology were published in 2015 as a result of the collaboration of the following oncological scientific societies: Polish Society of Surgical Oncology, Polish Society of Oncology, Polish Society of Clinical Oncology and Polish Society for Parenteral, Enteral Nutrition and Metabolism (POLSPEN). They recommend the start of the nutritional intervention in similar scenarios and emphasise the maintaining of the proper nutritional intervention order (Fig. 1) [10]. The authors of the above-mentioned recommendations emphasise the role of the preparation of a patient for surgery, in accordance with the recommendations of the ERAS (Enhanced Recover After Surgery) [11], which include, above all, the maintenance of physical activity, discontinuation of smoking, and proper diet for a minimum of 14 days before the surgery.

### Type of preoperative nutritional intervention

Patients enrolled in the radical treatment due to malignant neoplasms of the digestive tract belong to a specific group of patients. Medical treatments in these individuals are often of the planned nature. The extent and specifics of the oncological treatment are the reasons for enrolled patients to be in a good or fairly good overall condition. What is more, their up-to-date health record should be known and all of the health burdens and accompanying illnesses are known and treated. This provides the ability to proper preparation for a possible nutritional intervention.

The perioperative period is a special time for systemic metabolism, especially the postoperative recovery processes are aggravating. The healing process will be responsible for negative nitrogen balance, acceleration of metabolism, increased gluconeogenesis and production of acute-phase proteins. In patients with malnutrition—with the lack of adequate supply of nutrients in this situation—the risk of complications increases.

Although the first line of the intervention—nutritional consultation as well as the fortification of a diet and oral nutritional support (ONS)—is not debatable, in a case of inability of undergoing an oral feeding, the choice of the way of administration in patients before a surgery may represent a serious clinical obstacle. The choice of food supply routes at the surgical wards is affected, besides the obvious indications (Fig. 1), by a number of factors arising from the clinical situation of a patient (e.g. severity of malnutrition, the type of the planned surgery, the presence of other medical conditions), organisational capabilities (e.g. availability of method and experience, eventuality of home feeding), and others (e.g. patient’s will). All of the above factors are the reason for different practices being carried out within different health centres.

Often, during everyday hospital practice, there is a need to choose between enteral and parenteral way of nutrition therapy.

### Parenteral nutrition

Parenteral nutrition has been used since the 1960s, and its origins are linked with the activity of Stanley Dudrick, who was a pioneer in the intravenous use of mixed nutrition. A period of widespread use of this route of administration lasted until the 1990s, when published scientific works [12] and recommendations have changed in favour of enteral nutrition.

An intravenous route of administration of nutrients causes fast improvement of the nitrogen balance, fast recovery from injuries and decreases the amount of general and, above all, infectious complications [13, 14]. Ward described the reduction of non-infectious complications in severely malnourished patients from 42 to 5% [14]. The reduction in the number of severe complications in malnourished patients is also described by other authors [13, 15, 16]. The biggest positive effect of the parenteral nutrition can be observed in severely undernourished patients—this is around 5% of the patients which are operated [3].

Parenteral nutrition is no longer conclusively important in patients who are average or well nourished. Ward noted that in these groups of patients, the use of intravenous routes of administration nutrients can result in an increased amount of mortality [14]. Many authors describe that there is no effect in parenteral nutrition in decreasing the number of perioperative mortality [13, 15–18].

A distinguished element of the intravenous therapy is the number of complications, which can be divided into associated with intravenous infusion, metabolic and psychological aspects.

Complications of intravenous access can be described as early complications which are associated with the provision of route of administration into a vein (pneumothorax, bleeding, puncture of artery, air embolusm, cardiac arrhythmias) or late complications (permeability, catheter infections, sepsis, venous thrombosis). It should be noted that early complications do not happen often and are relatively easy to recognise, and the correct care associated with venipuncture and catheter greatly reduces the risk of the most dangerous septic complications.

Metabolic complications are also divided into early and late complications. Early complications may be e.g. fluctuations in blood glucose (hyperglycaemia/hypoglycaemia), metabolic acidosis, electrolyte disturbances, hypertriglyceridemia, abnormal liver function, excess fluid and refeeding syndrome. In the vast majority, they can be easily identified and relatively easy to treat. What is more, the implementation of appropriate procedures and appropriate monitoring of the patient can be effectively protected against them in future. The late complications—metabolic bone disease and PN-associated liver dysfunction (PNALD)—progress after a long period of time and are not an issue in fed patients before a surgery.

Psychological problems during parenteral nutrition may affect acceptance of the treatment by a patient who must undergo proper care of intravenous injections and several hours of intravenous infusions. The importance of a conversation with patients who must be advised regarding such proceedings should be pointed out. The consent of a patient undergoing this type of therapy is an absolute condition.

The impact of parenteral nutrition probably does not only arise from improving nutritional status, because it is difficult to expect any changes in severely undernourished patients after 2 weeks of therapy. It is believed that preoperative parenteral nutrition (PN) may work by reducing insulin resistance, the same as in the case of preoperative carbohydrate supply [19, 20].

Nature of preoperative nutritional therapy: preoperative stay of patients in a hospital and monitoring them in a surgical ward environment, provides a possibility of effective protection against the opportunity of developing serious complications.

### Enteral nutrition

Enteral nutrition is currently the primary recommended way of nutritional therapy [6–10]. It started developing since the 1990s, shortly after the work showing the impact of eternal nutrition on treatment results, e.g. it has been proven to reduce postoperative mortality by 50–70% [21, 22] and perioperative complications by up to 50% [23, 24]. Enteral nutrition (EN), despite its flaws, shortens the hospital stay, has fewer complications and reduces costs compared with PN [14, 25, 26]. EN is not associated with the risk of infections and immunodeficiency to the same extent as PN is [13].

It is crucial to mention the potential complications related to EN, which are divided into mechanical, intestinal, metabolic and psychological. The mechanical complications include aspiration, patency problems and positioning of a catheter or probe. Among enteric complication, one can list the nausea, vomiting, diarrhoea, constipation and abnormal absorption. Intestinal problems are often related to the type of administered food mixtures and speed of supply and can concern, with varying intensification within the initial period, up to 30% of patients. The metabolic complications include the following: changes in the blood glucose level, electrolyte disturbances and refeending syndrome. They are relatively easily recognised in surgical wards. The category of psychological problems contains primarily a patient’s acceptance to the presence of the probe or micro-jejunostomy as well as the potential intestinal complications.

The impact of EN on the body is highly extensive. In addition to providing nutrients, the maintenance of the natural way of nutrient absorption helps the intestinal mucosa retention, bile salt management and the secretion of gastrin. What is more, the stimulation of peristalsis may reduce the presence of pathogenic bacteria in the intestinal lumen. EN also stimulates the intestinal flow of blood, supports the immune system and the production of immunoglobin A, affects Th2 CD4 lymphocyte proliferation and prevents the proinflammatory effect of lymphocytes Th1 and the production of proinflammatory cytokines (IL-1, TNF-b).

A separate issue is the effect of the immunomodulatory agents. Despite the incomplete research, it seems that EN with the addition of immunomodulatory agents reduces the likelihood of all complications as well as the infectious complications and may shorten the length of hospital stay [27].

In many cases, EN could be carried out in a domestic environment, before surgery, especially in patients requiring preoperative therapy—e.g. preoperative radiochemotherapy in the case of locally advanced low oesophageal squamous cell cancer cT3/4cN+cM0.

### EN versus PN

Choice of the type of nutritional therapy in patients cannot be made without considering the advantages and disadvantages of each method (Table 2).

**Table 2:** The comparison of PN and EN in preoperative therapy

	PN	EN
Advantages	Easiness	Impact on the immune system
	Speed	Physiological way of delivery
	Efficiency	Safety
Disadvantages	Lack of recommendations	Therapy time, less calorific value
	High costs	Organisational
	Complications	Complications

Unmistakably, the use of EN reduces the number of perioperative complications and reduces time spent in a hospital [15, 17, 18]. By comparing the PN and EN, administrating EN reduces the risk of infectious complications; PN is associated with a greater risk of death and postoperative incidents in patients who are severely ill [15]. It needs to be remembered that it is extremely important to continue the nutritional intervention after the surgery [28].

Due to the lower calorific value of food mixtures and problems that may occur with the intake of nutrients by physiological route, an optimal intake by enteral route may be difficult, and fulfiled satisfaction of requirements takes more time than the intravenous route [29]. The way to optimise the supply can be a combination of EN and PN. Heidegger compared the effect of enteral and combination of enteral and parenteral therapies (SPN, supplemental parenteral nutrition) in 308 patients in ICU. The therapy showed that on the fourth day, the patients with EN received only 77% of energy needed, compared to 103% of the energy level in patients fed by the SPN therapy. He noticed fewer infections (27 from 38%) in the SPN and put forth a proposal that optimal coverage may have an impact on the reduction of hospital infections [30].

The cost of the PN is much higher. Braga calculated the difference between the two ways of conducting a therapy on $65; Bozetti’s calculation was $31 [26]. In Poland, the difference, in the refund from NFZ (the National Health Fund), between EN and PN is from 0 to around $50, depending on the intravenous therapy. The cost of preparations varies between around $15 to $85.

Patients who were prepared to scheduled surgery on the grounds of the gastrointestinal cancer, as a result of the frequency of malnutrition occurrence, often require nutritional intervention. In these patients, the nutrition therapy may be carried out in the hospital ward, which gives the possibility of suitable access to food and an option to monitor patients. In the case of hospitalised patients who overstay in a hospital, the cost is higher and may delay the start of oncological treatment. In view of the above arguments, it appears that the PN is preferably associated with oral or enteral administration of nutrients which gives an option to promptly and safely level up the nutritional deficiencies. It may be used in patients with malignant disease before the surgery.

## Conclusions

Enteral nutrition (EN) should be consider as a first treatment, in the case of nutritional intervention in patients before surgical treatment. There is no explicit evidence for the routine use of PN before every surgical treatment. However, it is known that a properly conducted PN is the secure method of supplementing malnutrition. Before the surgical treatment, assuming the strict observance of the personalisation of proceedings, patients may have been provided with parenteral nutrition; if possible, it should be combined with nutrients administration via the digestive tract.

## Abbreviations

EN: Enteral nutrition
ERAS: Enhanced Recover After Surgery
ESPEN: The European Society for Clinical Nutrition and Metabolism
ONS: Oral nutritional support
PN: Parenteral nutrition
POLSPEN: Polish Society of Clinical Oncology and Polish Society for Parenteral, Enteral Nutrition and Metabolism
SPN: Supplemental parenteral nutrition
